# Comparison of hysterosalpingograms with laparoscopy in the diagnostic of tubal factor of female infertility at the Yaoundé General Hospital, Cameroon

**DOI:** 10.11604/pamj.2015.22.264.8028

**Published:** 2015-11-19

**Authors:** Jean Dupont Kemfang Ngowa, Jean Marie Kasia, NGuefack-Tsague Georges, Victorine Nkongo, Charles Sone, Emmanuel Fongang

**Affiliations:** 1 Obstetrics and Gynecology Unit, Yaoundé General Hospital, P.O. Box 5408, Yaoundé, Cameroon; 2 Department of Obstetrics and Gynecology, Faculty of Medicine and Biomedical Sciences, University of Yaoundé I, P.O. Box 1364, Yaoundé, Cameroon; 3 Biostatistics Unit, Department of Public Health, Faculty of Medicine and Biomedical Sciences, University of Yaoundé I, P.O. Box 1364, Yaoundé, Cameroon; 4 Radiology Unit, Yaoundé General Hospital, P.O. Box 5408, Yaoundé, Cameroon

**Keywords:** Laparoscopy, hysterosalpingography, infertility, adnexal adhesion, tubal patency

## Abstract

**Introduction:**

The objectives were to assess the diagnostic value of hysterosalpingography (HSG) with laparoscopy as gold standard in the evaluation of tubal patency and pelvic adhesions in women suffering from infertility.

**Methods:**

We conducted a comparative cross sectional study on 208 medical files of infertile women followed up at the Yaoundé General Hospital during a period of five years (December 2007 to December 2012). Tubal patency, hydrosalpinx and pelvic adhesions detected at HSG were compared with laparoscopic findings as the gold standard. The sensitivity, specificity, positive predictive value (PPV), negative predictive value (NPV) and diagnostic accuracy of HSG were calculated with 95% confidence interval (CI).

**Results:**

Mean age of the patients was 31.4± 6.45 years. Secondary infertility was the most frequent type of infertility (66.82%). HSG had a moderate sensitivity (51.0%; 95% IC. 37.5-64.4), high specificity (90.0%; 95% IC.74.4-96.5), high PPV (89.3%; 95% IC. 72.8-96.3) and a moderate NPV (52.9%; 95% IC. 39.5-65.9) in the diagnosis of bilateral proximal tubal occlusion. Concerning, distal tubal patency, HSG had a high sensitivity (86.8%; 95% IC. 76.7-92.9), low specificity (42.2%; 95% CI. 29.0-56.7), moderate PPV (69.4%; 95% IC. 58.9-78.2) and a moderate NPV (67.9%; 95% IC. 49.3-82.0) in the diagnosis of bilateral or unilateral distal tubal occlusion. However, HSG had a low diagnostic value (27.8%; 95%IC.18.8-39.0) in the pelvic adhesions.

**Conclusion:**

HSG is of limited diagnostic value in tubal factor infertility and is of low diagnostic value for pelvic adhesions.

## Introduction

One of the most common and underappreciated reproductive health problems in developing countries is the high rate of infertility and childlessness [1, 2]. The inability to procreate is frequently considered a personal tragedy and a curse for the couple, impacting on the entire family and even the local community [3]. Tubo-peritoneal factors are responsible for about 30-40% of cases of female infertility and hence evaluation of tubal patency represents a key step and a basic investigation in the assessment of infertile women [4, 5]. Tubal occlusion is the most common underlying cause of infertility [6, 7]. In Africa Tubal factor infertility ranges from 42 to 77% in the literature [8]. Hysterosalpingography (HSG), laparoscopy with chromopertubation or both can be used to evaluate tubal patency. Owing to its noninvasive nature and low cost, HSG is widely used as a first-line approach to assess tubal patency and uterine anomalies in routine fertility workup [9, 10]. However, laparoscopy with chromopertubation has been the gold standard for investigating tubal patency [10]. The aim of this study was to compare hysterosalpingograms to laparoscopy as gold standard in the diagnosis of tubal factors of female infertility at the Yaoundé General Hospital in Cameroon, in order to determine their diagnostic value in our context.

## Methods

This was a comparative cross-sectional study based on medical records of 208 women followed up for infertility at the Obstetrics and gynecology unit of the Yaoundé General Hospital (YGH) in Cameroon from December 2007 to December 2012.We included medical records of infertile women investigated by HSG and laparoscopy during the study period for assessment of tubal patency and pelvic adhesions. We had beforehand obtained approval from the medical committee of the YGH to conduct this study. All HSGs were performed at the radiology unit on an outpatient basis between the 7th to the 10th day of menstrual cycle. A water soluble contrast medium was used. X-Ray Photographs were taken at the instant. Images were taken at the instant when the uterine cavity and tubes were filled with opaque material and when an overflow was seen at both sides of the tubes or when maximal filling of the tubes was observed without any overflow. After 30 minutes, a late film was made to assess the contrast material diffusion. HSG findings were classified as having no tubal occlusions, one-sided or bilateral proximal or distal tubal occlusion. The presence of hydrosalpinx or pelvic adhesions were also noted. Additional abnormalities of the uterine cavity were recorded as well. A diagnostic and/or operative laparoscopy was performed in the operating theatre under general anesthesia, during the follicular phase of the menstrual cycle before the ovulatory period. During the laparoscopy, inspection of the pelvis (genital organs) and the liver was performed, followed by testing for tube patency using methylene blue injected through the cervix via a Novak cannula. The presence of adhesions, structural abnormalities of the uterus, endometriosis and fallopian tube patency were sought for. Tubal patency assessed during laparoscopy was classified as no tubal occlusion, one-sided or two-sided proximal or distal tubal occlusion. When it was necessary, operative laparoscopy was performed. Data were entered and analyzed using IBM-SPSS Version 20 (Armonk, New York: IBM Corp.). Proximal and distal tubal occlusions, hydrosalpinx and pelvic adhesions diagnosed at HSG were compared with tubal occlusions, hydrosalpinx and pelvic adhesions detected at laparoscopy as gold standard. The sensitivity, specificity, positive predictive value (PPV), negative predictive value (NPV) and performance accuracy of HSG were calculated with 95% confidence interval.

## Results

Two hundred and eight women with a history of infertility who performed HSG and laparoscopy in their work up were included in this study. Table 1 shows the general characteristics of these patients. The mean age of the patients was 31.4± 6.4years (range from 19 to 44years). Secondary infertility was more frequent (66.82%) than primary infertility (28.36%), and married women were more represented (59.6%). Table 2 shows the performance of HSG in the diagnosis of tubal patency and pelvic adhesions compared to laparoscopy as gold standard. There was a moderate sensitivity (51.0%; 95% IC. 37.5-64.4) and a high specificity (90.0%; 95% IC.74.4-96.5) of HSG in the diagnosis of bilateral proximal tubal occlusion. However, there was a high positive predictive value (89.3%; 95% IC. 72.8-96.3) and a moderate negative predictive value (52.9%; 95%IC. 39.5-65.9) of HSG in the diagnosis of bilateral proximal tubal occlusion. This means that, only half of patients with bilateral proximal tubal occlusion and almost all patients with bilateral proximal tubal permeability are detected at HSG. However, when HSG revealed bilateral proximal tubal occlusion, there was about 90% probability that the tubes are really obstructed and when HSG demonstrated bilateral proximal tubal permeability, there was about 50% probability that the tubes were really patent. Concerning distal tubal patency, HSG had a high sensitivity (86.8%; 95%IC. 76.7-92.9) and a low specificity (42.2%; 95% CI. 29.0-56.7) in the diagnosis of bilateral or unilateral tubal occlusion. However, HSG had a moderate positive predictive value (69.4%; 95% IC. 58.9-78.2) and a moderate negative predictive value (67.9%; 95%IC. 49.3-82.0). This means that about 87% of patient with distal tubal occlusion (one or two sided) are detected by HSG and only 40% of patients with distal tubal permeability are diagnosed at HSG. Conversely, HSG had a high sensitivity (77.4%; 95%IC. 60.2-88.6) and a moderate specificity (61.1%; 95% CI. 47.8-73.0) in the diagnosis of hydrosalpinx. On the other hand, HSG had a low sensitivity (24.6%; 95% IC. 15.5-36.7) and specificity (45.4%; 95% IC. 21.3-72.0) in the diagnosis of pelvic adhesions.

**Table 1:** General patient characteristics

variables	Patients ; N=208	
	n	%
**Age range (years)**		
15-25	18	8.65
26-35	129	62.01
36-45	61	29.32
**Profession**		
House wife	82	39.42
worker	102	49.03
Student	24	11.53
**Marital Status**		
single	83	39.90
Married	124	59.6
**Type of infertility**		
Primary infertility	59	28.36
Secondary infertility	139	66.82

**Table 2:** Performance of HSG in diagnostic of tubal patency and pelvic adhesions compared to laparoscopy as gold standard

Factor of comparison*	TPR n (%)	FPR n (%)	FNR n (%)	TNR n (%)	Sensitivity % (95%CI)	Specificity % (95%CI)	PPV % (95%CI)	NPV % (95%CI)	Diagnostic Accuracy %(95%CI)
**Proximal tubal patency**									
Two-sided or One-sided occlusion	62 (44.0)	17 (12.0)	35 (24.8)	27 (19.1)	63.9 (54.0-72.8)	61.4 (46.6-74.3)	78.5 (68.2-86.1)	43.6 (54.9-70.6)	63.1 (54.9-70.6)
Two- sided occlusion	25 (31.6)	3 (3.8)	24 (30.4)	27 (34.2)	51.0 (37.5-64.4)	90.0 (74.4-96.5)	89.3 (72.8-96.3)	52.9 (39.5-65.9)	65.8 (54.9-75.3)
One-sided occlusion	12 (18.8)	14 (21.9)	11 (17.2)	27 (42.2)	52.2 (33.0-70.8)	65.9 (50.5-78.4)	46.2 (28.8-64.5)	71.0 (55.2-83.0)	60.9 (48.7-71.9)
**Distal tubal patency**									
Two-sided or One-sided occlusion	59 (52.2)	26 (23.0)	9 (8.0)	19 (16.8)	86.8 (76.7-92.9)	42.2 (29.0-56.7)	69.4 (58.9-78.2)	67.9 (49.3-82.0)	69.0 (60.0-76.8)
Two-sided occlusion	18 (32.7)	11 (20.0)	7 (12.7)	19 (34.5)	72 (52.4-85.7)	63.3 (45.5-78.1)	62.0 (44.8-77.3)	73.0 (53.9-86.3)	67.3 (54.1-78.2)
One-sided occlusion	10 (21.7)	15 (32.6)	2 (4.3)	19 (41.3)	83.3 (55.2-95.3)	55.9 (39.4-71.1)	40.0 (23.4-59.3)	90.5 (71.1-97.4)	63.0 (48.6-75.5)
Pelvic adhesions	15 (20.8)	6 (8.3)	46 (63.9)	5 (6.9)	24.6 (15.5-36.7)	45.4 (21.3-72.0)	71.4 (50.0-86.2)	9.8 (4.3-21.0)	27.8 (18.8-39.0)
Hydrosalpinx	24 (28.2)	21 (24.7)	7 (8.2)	33 (38.8)	77.4 (60.2-88.6)	61.1 (47.8-73.0)	53.3 (39.1-67.1)	82.5 (68.0-91.2)	67.1 (56.5-76.1)

* TPR = True Positive Rate; FPR = False Positive Rate; FNR = False Negative Rate; TNR = True Negative Rate; PPV = Positive Predictive Value; PPV = Negative Predictive Value; 95%CI = 95% Confidence Interval

## Discussion

Exploration of the female genital tract is one of the essential elements of infertility assessment. Laparoscopy provides both a panoramic view of the pelvic reproductive anatomy and a magnified view of pelvic organs and peritoneal surfaces. It is generally accepted that, diagnostic laparoscopy is the gold standard in diagnosing tubal pathology and other intra-abdominal causes of infertility [10–13]. Hysterosalpingography is a frequently utilized diagnostic method in the assessment of tubal status and detection of intra uterine anatomical defects in infertility diagnostic workup. However, the inadequacy of HSG in determining the state of tubal patency, emphasizes the need for laparoscopy.Good reliability of HSG in the diagnosis of proximal tubal blockage would make laparoscopy unnecessary and justify rather selective salpingography or passage to in vitro fertilization [14–16]. The reported sensitivity and specificity differed between studies concerning tubal occlusions [4, 10, 13, 14, 17, 18].

This study showed that HSG has a moderate sensitivity (51.0%; 95% IC. 37.5-64.4) and a high specificity (90.0%; 95% IC.74.4-96.5) in the diagnosis of proximal tubal occlusion. Both false negative and positive results can be seen with HSG in the diagnosis of proximal tubal occlusion [17]. The false positive result could be explained by the fact that spasm of uterine muscles during HSG following the injection of the contrast product may constrict or occlude one or both fallopian tubes. Small plugs of material, usually thought to be mucus or protein debris, can also occlude the proximal tube(s) where it is very narrow within the uterus [19]. Another scenario resulting in the false-positive diagnosis of proximal tubal occlusion is when inadequate wedging of the cervical cannula allows leakage of contrast material into the vagina, thus interfering with generation of sufficient intra cavitary pressure and often leading to the misdiagnosis of tubal occlusion [20]. Dessole S. et al reported 60% bilateral tubal patency on second HSG procedure one month after a first HSG demonstrated bilateral proximal tubal occlusion, which goes in favor of the explanations above [21]. The false negative proximal tubal patency rate at HSG in our study was 47.05% (IC 95%. 33.15-61.40) in bilateral occlusion and 28.94% (IC 95%. 15.98 - 46.11) in unilateral occlusion. The false negative result can be explained by the fact that contrast intravasation into uterine and ovarian veins during HSG can sometimes be mistaken for tubal filling [22]. Another explanation of false negative proximal test may be the long period between the achievement of the HSG and laparoscopy which can explain the occurrence of tubal pathology at the laparoscopy although absent at HSG.

The distal tubal occlusion is accessible to surgical therapeutic procedures and can lead to the practice of operative laparoscopy to improve fertility and prevent in vitro fertilization for some patients [23]. In the present study, about 87% of patients with one sided or two sided distal tubal occlusion were diagnosed at HSG while only 42% of distal tubal permeability were detected at HSG with a moderate PPV (69.4%) and NPV (67.9%). There are also false negative and positive distal tubal occlusions at HSG. The false positive results may be explained by the fact that in the presence of peritubal adhesions, even though the tubes may be patent, focal contrast deposits can lead to the misinterpretation as distal occlusions [13]. Another explanation should be the faulty technique occurring while performing HSG. Insufficient pressure during uterine injection of contrast material due to vaginal reflux or the absence of the late radiographs for detection of pelvic diffusion of contrast material can lead to misdiagnosing as distal occlusion. On the contrary, the false negative tubal distal occlusion can be explained by the huge contrast intravasation into pelvic veins which can be misinterpreted as tubal patency with peritoneal diffusion of contrast material. Another explanation is that, in case of one sided tubal distal occlusion, the pelvic diffusion of contrast materiel from one side can be misinterpreted as two sided tubal patency. Peri-tubal adhesions are a significant cause of infertility in women, altering the normal anatomic relationship between ovarian fimbriae and ovary and interfering or preventing the normal capture and transport of the ovum [24]. In accordance with other authors [10, 13, 17, 18, 25]. We recorded a low sensitivity (24.6%) and specificity (45.4%) of HSG in diagnosing pelvic adhesions in this study. One of the limits of this study is that we didn't taken into consideration the possible variability of HSG interpretation among radiologists and the time interval between HSG and laparoscopy which could influenced the difference in the results of these two diagnostic tests. However, this study provided information on the diagnostic value of HSG in our setting.

## Conclusion

The results of this study reveal that Hysterosalpingography is of limited diagnostic value in tubal factor infertility and of low diagnostic value for pelvic adhesions. Therefore, we believe that laparoscopy should be performed in cases of abnormal hysterosalpingograms and even in cases of normal hysterosalpingograms in the context of unexplained infertility.
